# Bioactive Compounds from Endophytic Bacteria *Bacillus subtilis* Strain EP1 with Their Antibacterial Activities

**DOI:** 10.3390/metabo12121228

**Published:** 2022-12-07

**Authors:** Muhammad Numan, Muddaser Shah, Sajjad Asaf, Najeeb Ur Rehman, Ahmed Al-Harrasi

**Affiliations:** 1 Natural & Medical Sciences Research Center, University of Nizwa, Nizwa 616, Oman; 2 Department of Biology, University of North Carolina at Greensboro, Greensboro, NC 27412, USA; 3 Department of Botany, Abdul Wali Khan University Mardan, Mardan 23200, Pakistan

**Keywords:** secondary metabolites, *Bacillus subtilis* strain EP1, antibacterial activity, NMR spectroscopy, HR-ESI-MS

## Abstract

Endophytic bacteria boost host plant defense and growth by producing vital compounds. In the current study, a bacterial strain was isolated from the *Boswellia sacra* plant and identified as *Bacillus subtilis* strain EP1 (accession number: MT256301) through 16S RNA gene sequencing. From the identified bacteria, four compounds—**1** (4-(4-cinnamoyloxy)phenyl)butanoic acid), **2** (cyclo-(L-Pro-D-Tyr)), **3** (cyclo-(L-Val-L-Phe)), and **4** (cyclo-(L-Pro-L-Val))—were isolated and characterized by 1D and 2D NMR and mass spectroscopy. Moreover, antibacterial activity and beta-lactam-producing gene inhibition (δ-(l-α-aminoadipyl)-l-cysteinyl-d-valine synthetase (ACVS) and aminoadipate aminotransferase (AADAT)) assays were performed. Significant antibacterial activity was observed against the human pathogenic bacterial strains (*E. coli*) by compound **4** with a 13 ± 0.7 mm zone of inhibition (ZOI), followed by compound **1** having an 11 ± 0.7 mm ZOI. In contrast, the least antibacterial activity among the tested samples was offered by compound **2** with a 10 ± 0.9 mm ZOI compared to the standard (26 ± 1.2 mm). Similarly, the molecular analysis of beta-lactam inhibition determined that compounds **3** and **4** inhibited the two genes (2- to 4-fold) in the beta-lactam biosynthesis (ACVS and AADAT) pathway. From these results, it can be concluded that future research on these compounds could lead to the inhibition of antibiotic-resistant pathogenic bacterial strains.

## 1. Introduction

Endophytes are well-known to enhance the growth and development of host plants in diverse environmental circumstances [1,2]. The role of endophytic bacteria (EPB) in boosting the host plants’ resistance against phytopathogens and promoting biological nitrogen fixation can promote the growth of plants. These properties can also be exploited in agricultural practice [3]. Furthermore, the capability of endophytic bacteria to yield numerous bioactive constituents is important to devising novel drugs in the pharmaceutical industry [4]. To identify and explore endophytic bacteria, many researchers/scientists are involved in the isolation and screening of endophytes for various bioactive natural products. In the past few decades, several endophytic bacteria have been shown to produce natural products such as phytohormones, low molecular weight compounds, enzyme inhibitors, siderophores, and antibiotics [5,6].

The chemical constituents obtained from endophytic bacteria have promising properties as antibacterial agents to resist microbes, scavenge free radicals, and serve as an effective remedy to treat cancer and diabetes [7,8,9,10,11]. Therefore, unexplored endophytic bacteria, specifically from medicinal plants, need to be screened and analyzed for the presence of novel antimicrobial agents [12,13,14]. The human population is rapidly increasing, and due to anthropogenic activities, the ecosystem is affected, leading to multiple human health disorders [15]. Limited phytochemical information is available in the literature on how endophytes regulate their hosts by synthesizing primary and secondary metabolites and how these constituents affect the shape and growth of endophytic bacterial communities [16]. Therefore, the search for new antimicrobial drugs to deal with these resistant forms of TB has gained global attention, and researchers/scientists are searching for a novel source to subsist microbial complications [17,18,19,20,21].

Microorganisms, animals, and plants are also the prime sources of natural bioactive ingredients [8,22,23], such as taxol, an anticancer drug isolated from *Taxus wallichiana* [24]. Cyclosporine isolated from *Tolypocladium inflatum,* which serves as a classical immunosuppressive, proved the significance of endophytes [25]. Endophytic bacteria are also affluent sources of innovative antibiotics comprising ecomycins, pseudomycins, munumbicins, and kakadumycins [26]. In addition, some Mycobacterium strains have developed strong resistance to numerous first-line anti-tubercular medications, and a few second-line anti-tubercular medicines take the lead in multi-drug resistance (MDR) and other extensive drug resistance tuberculosis (XDR-TB) tubercle bacilli [27]. Munumbicin B has been observed with antibiotic significance isolated from the endophytic bacterium and is especially active against multi-drug resistant tuberculosis (MDR-TB) [26].

Due to the huge diversity and untapped potential of endophytic bacteria, they appear as an alternate source of bioactive natural products with potential applications in the pharmaceutical industry. Hence, phytochemical investigations of endophytic bacteria for the discovery of antibacterial drugs have taken much attention. Therefore, the current study was designed to explore further the significance of the endophytic bacteria by screening them for their active constituents and testing them against human pathogenic bacteria.

## 2. Materials and Methods

### 2.1. Bacterial Isolation and Purification

The bacteria were isolated from the samples, as earlier reported by Barzanti et al. [28], with slight modification. Both the leaves and stems of *Boswellia sacra* trees were selected to isolate the endophytic bacteria. The samples were correctly cleaned with autoclaved deionized distilled water. The samples were sterilized using sodium hypochlorite (5%) through a shaking incubator for 10 min at 120 rpm [29]. To isolate the bacterial endophytes, the samples’ chopped tissues (1 mm) were put into nutrient broth media plates. After 24 h, the prepared colonies were properly subcultured into new Petri plates.

### 2.2. DNA Extraction, Sequencing, and Identification

DNA from the isolated bacteria was extracted, as earlier documented by Numan et al. [30]. The genomic DNA was carefully removed from the 24-hour-old culture through a lysis buffer, ensued by polymerization of DNA via a thermocycler. After confirmation of DNA on Gel electrophoresis, it was purified through the “Thermo Scientific Gene JET Gel Extraction Kit”. The amplified bacterial DNA sequences through 16S rRNA were processed for sequencing to produce a gene sequence of 16S rRNA. The gene sequence was analyzed through the NCBI BLAST computer program and compared with the nucleotides on the gene bank database [31,32]. To determine the phylogenetic position and closely matching strains of isolate, the amplified sequence was compared with sequences obtained from NCBI through BLAST using the EzTaxon server (http://www.eztaxon.org/). Multiple sequence alignments of the test sequence were performed using CLUSTAL_X version 8.3. The phylogenetic tree was constructed by the maximum likelihood process through MEGA X software.

### 2.3. Extraction of Biomass

The secondary metabolites were extracted following the Li et al. [33] method, with slight modification. Briefly, bacteria were inoculated in a growth media for seven days at 30 °C while their colony-forming unit (CFUs) were regularly monitored through OD600 nm. Once maximum growth (OD600 nm value 2.5) was achieved, extraction was started by centrifuging the culture at 10,000 (*g* × force) for 10 min, followed by its purification through an organic solvent ethyl acetate (EtOAc).

### 2.4. Extraction and Isolation of Pure Compounds

The whole biomass of (2.8 g) of *Bacillus subtilis* EP1 strain was extracted with ethyl acetate (EtOAc, 250 mL, three times), using a glass separating funnel, followed by continuous shaking, as earlier described by Raina et al. [34] with slight modifications. The EtOAc fraction (36.5 mg) was further passed through silica gel column chromatography (CC). Five fractions (Fr_1_-Fr_5_) were obtained using an increasing gradient solvent system of *n*-hexane, *n*-hexane/EtOAc, and pure EtOAc. Based on the thin layer chromatography (TLC) profile, three fractions Fr_2_ (8 mg), Fr_3_ (6 mg), and Fr_4_ (9.5 mg) (similar in chemical nature) were combined together and further subjected to CC affording compound **2** (10.5 mg, 60% EtOAc/*n*-hexane), **3** (8.5 mg, 40% EtOAc/*n*-hexane), **4** (7.0 mg, 30% EtOAc/*n*-hexane), and **1** (8.3 mg, 80% EtOAc/*n*-hexane). Structure elucidation of these compounds was conducted by extensive spectroscopic techniques, including 1D (^1^H and ^13^C) and 2D (HSQC, HMBC, and COSY) nuclear magnetic resonance (NMR) techniques, high-resolution electron spray ionization mass spectrometry (HR-ESI-MS), and also by comparison with the literature.

Colorless powder; UV (MeOH)λ*_max_*: 222, 280 nm; FT-IR (solid)υ_max_: 3430, 1735, 1693, 1630, 1594, 1526, 1220, 1020, 920 cm^−1^; HRMS (ESI+): *m/z* 334.11554 [M+H+Na]^+^ (calcd. 334.11810 (C_19_H_19_NaO_4_); ^1^H-NMR (CD_3_OD, 600 MHz): δ 7.78 (1H, d, *J* = 16.2 Hz, H-3′), 7.54 (2H, t, *J* = 7.2, 3.0 Hz, H-5′/9′), 7.39 (3H, dd, *J* = 7.2, 3.2 Hz, H-6′/8′, H-7′), 7.29 (2H, d, *J* = 7.8 Hz, H-6/10), 7.20 (2H, d, *J* = 7.8 Hz, H-7/9), 6.44 (1H, d, *J* = 16.2 Hz, H-2′), 2.97 (2H, t, *J* = 7.8 Hz, H-4), 2.69 (2H, t, *J* = 7.8 Hz, H-2), 1.23 (2H, m, H-3); ^13^C-NMR (CD_3_OD, 125 MHz): δ 178.3 (C-1), 171.8 (C-1′), 157.3 (8), 147.1 (C-3′), 140.2 (C-5), 134.0 (C-4′), 130.8 (C-5′/9′), 129.0 (C-6′/8′), 128.6 (C-7′), 128.4 (C-6, 128.3 (C-10), 126.4 (C-7/9), 117.2 (C-2′), 35.5 (C-4), 30.6 (C-2), 29.7 (C-3).

### 2.5. Antibacterial Activities

Antibacterial activity was evaluated against the *Escherichia coli* (*E. coli*) strain available at the Natural and Medical Sciences Research Center at the University of Nizwa Oman. The *E. coli* strain was grown in the nutrient broth media until the optical density (OD600) reached 0.5. The culture was spread on the nutrient agar plates, and the compounds at different concentrations (1 mg/mL, 500 µg/mL, 250 µg/mL, 100 µg/mL, 50 µg/mL, and 10 µg/mL) were applied by disc diffusion method [35]. After 24 h, the zones of inhibition were measured, and pictures were taken.

### 2.6. Molecular Validation of the Antibacterial Activities

#### 2.6.1. RNA Extraction

RNA was extracted from four days old bacterial strains grown in nutrient broth media following the technique as stated by Ríos-Marco et al. [35]. The cells (100 mg) were washed twice with DPEC-treated autoclaved distilled water and transferred to RNAase/DNase-free Eppendorf tubes. Moreover, 0.5 mL Trizol and 200 µL chloroform were added and homogenized by vortexing for 30 s, followed by incubation at 4 °C for 15 min and centrifugation (12,000× *g* for 15 min at 4 °C). The aqueous phase was shifted to a new tube with further addition of 0.5 mL isopropanol and then incubated for 10 min on ice. The samples were centrifuged at 12,000× *g* for 10 min at 4 °C. The supernatant was removed, and the remaining pellet was washed with 1 mL of 70% ethanol, centrifuged, and air-dried at room temperature for 10 min. The isolated RNA was re-suspended in 50 µl RNAase-free water.

#### 2.6.2. cDNA Synthesis and QRT PCR Analysis

For cDNA synthesis and QRT PCR, the Field [36,37] methods were slightly modified. RNA of 100 ng/µL was used for cDNA synthesis. Master Mix was prepared using 25x dNTPs, Reverse transcriptase enzyme, 10 × RT Random primers, and nuclease-free water. RNA was added to the Master Mix according to its concentration, such as for each 100 ng/µL RNA, 10 µL was taken for the cDNA synthesis. The polymerase chain reaction was carried out in a thermo-cycler at 25 °C for 10 min, 37 °C for 2 h, and 85 °C for 5 mins. The synthesized cDNA was refrigerated at −80 °C until further use.

The synthesized cDNA was utilized for the gene’s amplification presented in Table 1. Ubiquitin was used as a housekeeping gene. Power up “SYBR” green Master Mix (applied biosciences thermoscienfic) was employed for the thermo-cycler (Quant Studio 5 by applied biosystems life technologies) PCR reaction; 10 pM primers (forward and reverse) were utilized for all genes. The reaction was accomplished in triplicate to minimize errors and contaminations. The reaction was conducted at a temperature of 94 °C for 10 min in stage 1, 40 cycles of PCR reaction at 94 °C for 45 s, 65 °C for 45 s, 72 °C for 1 min, and eventually, the extension temperature was set at 72 °C for 7 min. The threshold level of 0.1 was set for gene amplifications. Gene expression findings were evaluated via delta-delta CT calculation techniques in Microsoft Excel according to the Schmittgen and Livak method [38]. Fold change in gene expression analysis was estimated using the following formula:

(1) $$\mathrm{Fold}\;\mathrm{change}=2^{-∆∆\mathrm{CT}}$$

where $2^{-∆∆\mathrm{CT}}=\left[\left(C_{T}\;\mathrm{the}\;\mathrm{gene}\;\mathrm{of}\;\mathrm{interest}-{\;C}_{T}\;\mathrm{internal}\;\mathrm{control}\right)\;\mathrm{Sample}\;A-\left(C_{T}\;\mathrm{the}\;\mathrm{gene}\;\mathrm{of}\;\mathrm{interest}-C_{T}\;\mathrm{internal}\;\mathrm{control}\right)\;\mathrm{Sample}\;B\right)].$

Graph-pad prism (v10.1) with ANOVA analysis was used for graphical analysis and statistic calculations. The data is presented with mean values of three replications.

## 3. Results and Discussion

### 3.1. Strain Identification

**Figure 1:**
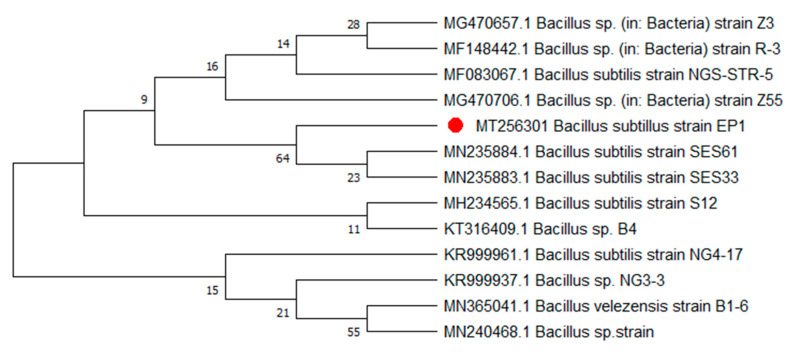
Phylogenetic tree of the isolated strain *B. subtilis* strains EP1. The strain is highlighted by the red bullet. The tree was constructed using maximum likelihood with bootstrap values (1000).

Colony color, size, odor, shape, and symmetry were identified. Medium size, pink colonies were observed on nutrient broth media plates. Phylogenetic analysis of the isolate identified in the current screening depicted that the isolate belongs to the *Bacillus* genera (Figure 1).

### 3.2. Characterization of Compounds through NMR and Mass Spectroscopy

**Figure 2:**
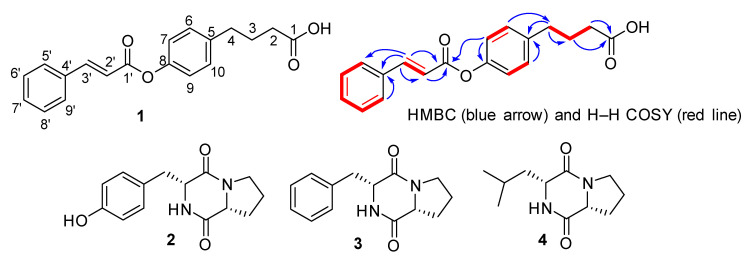
Structure of the new (**1**) and known compounds (**2–4**) isolated from bacterial strain.

Bioassay-guided isolation of ethyl acetate extract of bacterial strain resulted in the isolation of three cyclic dipeptides; cyclo-(L-pro-D-tyr) **2** as earlier stated by Wattana-Amorn et al. [41], cyclo-(L-val-L-phe) **3** and cyclo-(L-pro-L-val) **4** as reported by Alshaibani et al. [42] and Al-Hosni et al. [43], and one new benzoic acid known as 4-(4-cinnamoyloxy)phenyl) butanoic acid **1** (Figure 2). The dipeptides were isolated from this bacterial strain for the first time, while compound **1** was isolated as a new natural product.

Compound **1**, obtained as a colorless amorphous powder, was studied to have the molecular formula of C_19_H_18_O_4_, as identified by HR-MS at *m/z* = 332.9645 [M + Na]^+^. Its UV spectrum showed absorption bands at λ*_max_* 222 and 280 nm, and its IR spectrum revealed absorption bands at υ_max_ 3430 (hydroxyl), 1735 (ester), 1693 (carboxylic acid), 1630 (vinyl C=C), and 1594, 1526, 1220, 1020 cm^−1^ (aromatic ring). Analysis of ^1^H-NMR spectrum and correlation with ^13^C-NMR spectra exhibited two aromatic A_2_B_2_ systems involving one *para*-substituted benzene ring (C-5 to C-10) at δ_H_ 7.54 (2H, t = 7.2, 3.0 Hz) and 7.39 (3H, dd, *J* = 7.2, 3.2 Hz), and one monosubstituted benzene ring (C-4′ to C-9′) at δ_H_ 7.29 (2H, d, *J* = 7.8 Hz) and 7.20 (2H, d, *J* = 7.8 Hz). Two sp^2^ methine signals in the ^1^H-NMR spectra at 7.78 (1H, d, *J* = 16.2 Hz, H-3′) and 6.44 (1H, d, *J* = 16.2 Hz, H-2′) and their HMBC correlation with C-1′ (δ_C_ 171.8), proved the presence of cinnamic acid (Figure 2). The ^13^C-NMR and DEPT spectra of compound EPC-1 showed the presence of nineteen carbon signals, including nine aromatic methines (δ_C_ 130.8, 130.8, 129.0, 129.0, 128.6, 128.4, 128.3, 126.4, 126.4), two aliphatic methines (δ_C_ 147.1, 117.2), three methylenes (δ_C_ 35.5, 30.6, and 29.7), and five quaternary carbons (δ_C_ 178.3 (carboxylic acid), 171.8 (ester), 147.1, 140.2, and 134.0).

The HMBC correlation of H-2′ and H-3′ with C-4′ (δ_C_ 134.0) confirmed the attachment of double bond conjugation with the aromatic A_2_ system. The HMBC correlation of H-7 (δ_H_ 7.20) with C-8 (δ_C_ 147.1) and C-1′ (δ_C_ 171.8) further confirmed the attachment of oxygen at the *para* position of the B_2_ aromatic ring. Three signals at δ_H_ 2.97 (2H, t, *J* = 7.8 Hz), 2.69 (2H, t, *J* = 7.8 Hz), and 1.23 (2H, m) in the ^1^H-NMR spectrum showed the presence of three methylenes, which were further confirmed by the DEPT 135 experiment. The ^1^H-^1^H COSY correlation between H-2, H-3, and H-4 indicated that the methylenes are in a straight chain. The HMBC correlation of H-2 and H-3 with C-1 (δ_C_ 178.3) showed the presence of a carboxylic group at one end of the chain. In contrast, the HMBC correlation of H-4 with C-5 (δ_C_ 140.2) and C-6 (δ_C_ 128.4) confirmed the attachment of another side of the straight chain at the para position of the B_2_ aromatic system (Figure 2). Based on these combined spectroscopic techniques, the structure of **1** was concluded and named 4-(4-cinnamoyloxy)phenyl)butanoic acid.

### 3.3. Antibacterial Significance

The antibacterial significance of isolated compounds was examined against the human pathogenic strain *E. coli.* All the compounds displayed considerable potential at the concentration of 1 mg/mL (Figure S1). Furthermore, compound **4** offered maximum activity against the *E. coli* with a 13 ± 1 mm zone of inhibition (ZOI), followed by compound **1** with an 11 ± 1 mm ZOI and compound **3** having an 11 ± 0.5 mm ZOI, while the least activity was presented by compound **2** with 10 ± 1 mm, compared to the standard with a 26 ± 1 mm ZOI at a concentration of 10 µg/mL. Furthermore, the antibacterial screening revealed that ZOI increases with an increase in the concentration of the compounds. However, the DMSO also inhibited the *E. coli* strain but was negligible to consider (Table 2).

Compounds **2**–**4** were isolated previously from Streptomyces SUK 25 and screened against several MDR pathogens, such as *Enterococcus raffinosus*, *Staphylococcus aureus*, *Klebsiella pneumoniae*, *Acinetobacter baumanii*, *Pseudomonas aeruginosa*, and *Enterobacter spp.* [42]. Compound **3** did not show any activity, while compound **2** was active only against *Enterococcus raffinosus* with the ZOI at 13 mm. Both compounds were recently reported from the deep sea-derived *Streptomyces xiamenensis* MCCC 1A01570. They showed mild in vitro cytotoxicity against three cancer cell lines (ECA-109, Hela-S3, and PANC-1), with the inhibition rates arranged from 50% to 65% [43].

Compound **2** was previously isolated from *Streptomyces* sp. strain 22-4 and found to be active against *Xanthomonas axonopodis pv. citri* and *Ralstonia solanacearum* with MIC of 31.25 μg/mL. No activity was observed against *Clavibacter michiganensis* [40]. Abed et al. [44] isolated compound **2** from a hypersaline cyanobacterial material collected from wadi Muqshin in southeastern Oman, reduced quorum-sensing-dependent luminescence of the reporter *E. coli* pSB401 induced by N-3-oxohexanoyL-L-homoserine lactone [40]. The same compound was reported from *Bacillus* sp. N strain [45] and *Bacillus cereus subsp. thuringiensis* [46], associated with a rhabditid entomopathogenic nematode with prominent antimicrobial activities.

**Figure 3:**
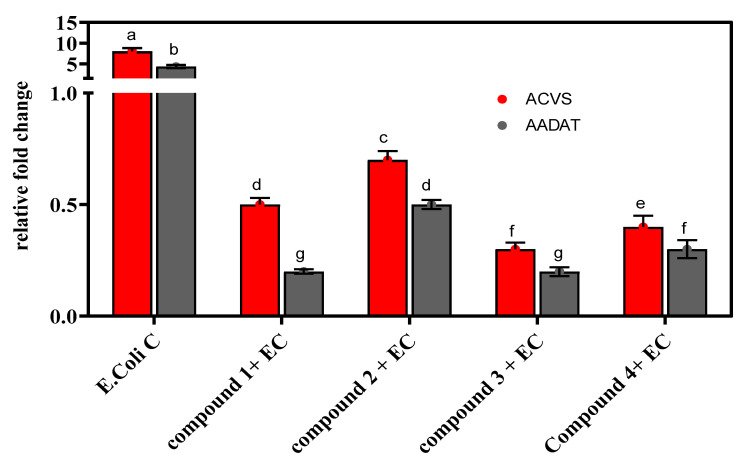
Inhibition of beta-lactam gene (δ-(l-α-aminoadipyl)-l-cysteinyl-d-valine synthetase (ACVS) and aminoadipate aminotransferase (AADAT)) in *E. coli* by compounds isolated from bacteria. EC = *E. coli*. DMRT test was performed for statistically significant variations. Small letters (a to g) show variation across different treatments.

Compound **1** was isolated as a new natural product never reported before, has depicted resistance to the tested bacterial strain (Table 2), and was observed to inhibit the beta-lactam pathway (Figure 3). However, compound **1** is less active against the pathogenic strain compared to the other compounds. This is a novel compound and has not been assessed previously for any antimicrobial activity. This study is the first ever to report the antibacterial activity of this compound against *E. coli*. Further research is required to fully understand the antimicrobial role of this compound. Compound **2** exhibited considerable potential against the tested bacterial strains. Thus, our data agreed with previously reported outcomes for antibacterial activity [40] and antifungal activities [47]. Our current study revealed that the compounds were effective against the human pathogenic strain *E. coli* (Table 2). The activity of compounds against the bacteria increased with the dosage concentration.

### 3.4. Beta-Lactam Gene Inhibition

The compound’s activity was assessed for inhibiting two precursor genes (ACVS and AADAT) for the beta-lactam pathways. It can be observed that in the control sample, both genes were expressed at a fold change of around 8 (Figure 3). However, when the *E. coli* strain was treated with the isolated compounds, it suppressed the expression of these two genes. These compounds have significantly suppressed the expression of ACVS and AADAT genes compared to control samples. For example, the *E. coli* strain treated with compound **1** has reduced the expression of these genes up to 7.5 folds compared to the control sample. Furthermore, similar findings were noticed for the other three compounds that reduce the expression of the beta-lactam pathway genes up to 7 to 7.7 folds as compared to control samples, where the expression is around 8 folds.

These compounds were also tested for the inhibition of beta-lactam pathway genes and were found to inhibit the synthesis of the beta-lactam pathway mildly. Compound **3** has already been reported for antibacterial and anticancer activities [43,48]. Gos et al. [49] reported this compound (cyclo-(L-Val-L-Phe)) from endophytic actinomycetes and was found to be active against pathogenic bacteria. In our study, we used the same compound against the *E. coli* strain for bactericidal activity and to inhibit the biosynthesis of beta-lactam by suppressing the genes for the beta-lactam pathway (ACVS and AADAT). It showed moderate activity against *E. coli* by the disc diffusion method. The activity tended to increase when concentration was increased and vice versa. However, it inhibited the beta-lactam biosynthesis by suppressing the precursor genes for the beta-lactam pathway. Similarly, compound **4** also presented the reported microbial activity documented by Nishanth Kumar et al. [50]. We performed the antibacterial activity on this compound against the human pathogenic strain *E. coli* and found that it determined good antibacterial activity against this strain. As mentioned for the other compounds, the activity of compound **4** was also dose dependent. Moreover, the beta-lactam inhibition was performed with two beta-lactam pathway genes (ACVS and AADAT). Compound **4** mildly suppressed the expression of these genes, which reflects the inhibition of beta-lactam biosynthesis. These results determined that bacteria are a vital source of the compounds responsible for bactericidal activities and could be used against bacteria resistant to common drugs and antibiotics.

## 4. Conclusions

In the present study, one new (**1**) and three known compounds (**2–4**) were isolated from an endophytic bacterial strain *B. subtilis* strain EP1. All the isolated compounds offered moderately good antibacterial potential against the *E. coli* strain from low to high doses. Compound **4** presented the highest promising potential to inhibit the *E. coli* among the tested compounds. The compounds were also found to inhibit beta-lactam biosynthesis by inhibiting the beta-lactam genes (ACVS and AADAT). Compounds **3** and **4** were found to inhibit these genes up to 4-fold as compared to the control sample. Further studies are considered necessary to isolate some more bioactive constituents and screen them for antibiotic-resistant bacteria.

## Figures and Tables

**Table 1:** Primers of the beta-lactam pathway genes (δ-(l-α-aminoadipyl)-l-cysteinyl-d-valine synthetase (ACVS) and aminoadipate aminotransferase (AADAT)) used for qRT PCR analysis.

Beta-Lactam Biosynthesis			
Gene Name	Code	F	Reference
δ-(l-α-aminoadipyl)-l-cysteinyl-d-valine synthetase	ACVS F	AGCCGTGAAAAGCCACTTGA	[39]
	ACVS R	GCTCACTGATGCCAATGGTTC	
aminoadipate aminotransferase	AADAT F	GTGGCATCGGTAGAGAAGCATT	
	AADAT R	CAACGTGAAGCATTCTCATCCA	
Ubiquitin	UBQ F	CAATCACCTTGGAAGTCGAGTCGTC	[40]
	UBQ R	CTGGATGTTGTAGTCGGAAAGGGTG	

**Table 2:** Antibacterial activity of four compounds isolated from *B. subtilis* strain EP1 against *E. coli*.

	1	2	3	4
Concentration	(Mean ± SD in mm)			
Amoxicillin	24 ± 1	23 ± 1	20 ± 1	26 ± 1
1 mg/mL	11 ± 1	10 ± 1	11 ± 0.5	13 ± 1
500 µg/mL	9 ± 1	10 ± 0.5	10 ± 0.5	11 ± 0.5
250 µg/mL	9 ± 1	9 ± 0.5	9 ± 0.5	10 ± 0.5
100 µg/mL	7 ± 0.5	9 ± 1	10 ± 0.5	9 ± 0.5
50 µg/mL	8 ± 1	8 ± 0.5	8 ± 0.5	9 ± 0.5
10 µg/mL	7 ± 1	7 ± 0.5	7 ± 0.5	8 ± 0.5
DMSO	6 ± 0.5	6 ± 0.5	6 ± 0.5	6 ± 0.5

Data were taken in triplicates and measured in millimeters. SD = standard deviation, DMSO = Dimethyl sulfoxide.

## Data Availability

The data presented in this study are available in the article.

## Supplementary Materials

The following supporting information can be downloaded at: https://www.mdpi.com/article/10.3390/metabo12121228/s1. Figure S1: Antibacterial activity of the isolated compounds measured as disc diffusion; Figure S2: ^1^H-, ^13^C-NMR, and HR-ESI-MS spectrum of 1; Figure S3: 1H-, 13C-NMR, and HR-ESI-MS spectrum of **2**; Figure S4: ^1^H-, ^13^C-NMR, and HR-ESI-MS spectrum of **3**; Figure S5: ^1^H-, ^13^C-NMR, and HR-ESI-MS spectrum of **4**.

## Sample Availability

Samples of the compounds are not available from the authors.
