# Supplementary material for: Bioactive Compounds from Endophytic Bacteria Bacillus subtilis Strain EP1 with Their Antibacterial Activities
Source: Metabolites. 2022 Dec 7;12(12):1228. doi: 10.3390/metabo12121228 (PMC9788538; doi:10.3390/metabo12121228)
Supplement: Supplementary file 1 [file metabolites-12-01228-s001.zip › metabolites-1969285-supplementary.pdf]

Supporting Information

Bioactive Compounds from Endophytic Bacteria *Bacillus subtilis* strain EP1 with their Antibacterial Activities

Muhammad Numan [1,2], Muddaser Shah [1,3], Sajjad Asaf [1], Najeeb Ur Rehman [1,*] and Ahmed Al-Harrasi [1,*]

1 Natural & Medical Sciences Research Center, University of Nizwa, Nizwa 616, Oman; sajjadasaf@unizwa.edu.om (S.A.)

2 Department of Biology, University of North Carolina at Greensboro, NC 27412, USA; numanbiotech@gmail.com (M.N)

3 Department of Botany, Abdul Wali Khan University Mardan, Mardan 23200, Pakistan; muddasershah@awkum.edu.pk (M.S.)

*Correspondence: najeeb@unizwa.edu.om (N.U.R); aharrasi@unizwa.edu.om (A.A.-H)

Abstract: Endophytic bacteria boost host plant defense and growth by producing vital compounds. In the current study, a bacterial strain was isolated from the *Boswellia sacra* plant and identified as *Bacillus subtilis* strain EP1 (accession number: MT256301) through 16S RNA gene sequencing. From the identified bacteria, four compounds; **1** (4-(4-cinnamoyloxy)phenyl)butanoic acid), **2** (cyclo-(L-Pro-D-Tyr)), **3** (cyclo-(L-Val-L-Phe)), and **4** (cyclo-(L-Pro-L-Val)) were isolated and characterized by 1D and 2D NMR and mass spectroscopy. Moreover, antibacterial activity and beta-lactam-producing gene inhibition [(δ-(l-α-aminoadipyl)-l-cysteinyl-d-valine synthetase (ACVS) and aminoadipate aminotransferase (AADAT)] assays were performed. Significant antibacterial activity was observed against the human pathogenic bacterial strains (*E. coli*) by compound **4** with 13 ± 0.7 mm zone of inhibition (ZOI) followed by compound **1** having 11 ± 0.7 mm ZOI. In contrast, the least antibacterial activity among the tested samples was offered by compound **2** with 10 ± 0.9 mm ZOI compared to the standard (26 ± 1.2 mm). Similarly, the molecular analysis of beta-lactam inhibition determined that compounds **3** and **4** have inhibited the two genes (2 to 4-fold) in the beta-lactam biosynthesis (ACVS and AADAT) pathway. From these results, it can be concluded that future research on these compounds could lead to the inhibition of antibiotic-resistant pathogenic bacterial strains.

Keywords: Secondary metabolites; *Bacillus subtilis* strain EP1; antibacterial activity; NMR spectroscopy; HR-ESI-MS.

Citation: Lastname, F.; Lastname, F.; Lastname, F. Title. *Metabolites* **2022**, *12*, x. https://doi.org/10.3390/xxxxx

Academic Editor: Firstname Lastname

Received: date
Accepted: date
Published: date

Publisher's Note: MDPI stays neutral with regard to jurisdictional claims in published maps and institutional affiliations.

Copyright: © 2022 by the authors. Submitted for possible open access publication under the terms and conditions of the Creative Commons Attribution (CC BY) license (https://creativecommons.org/licenses/by/4.0/).

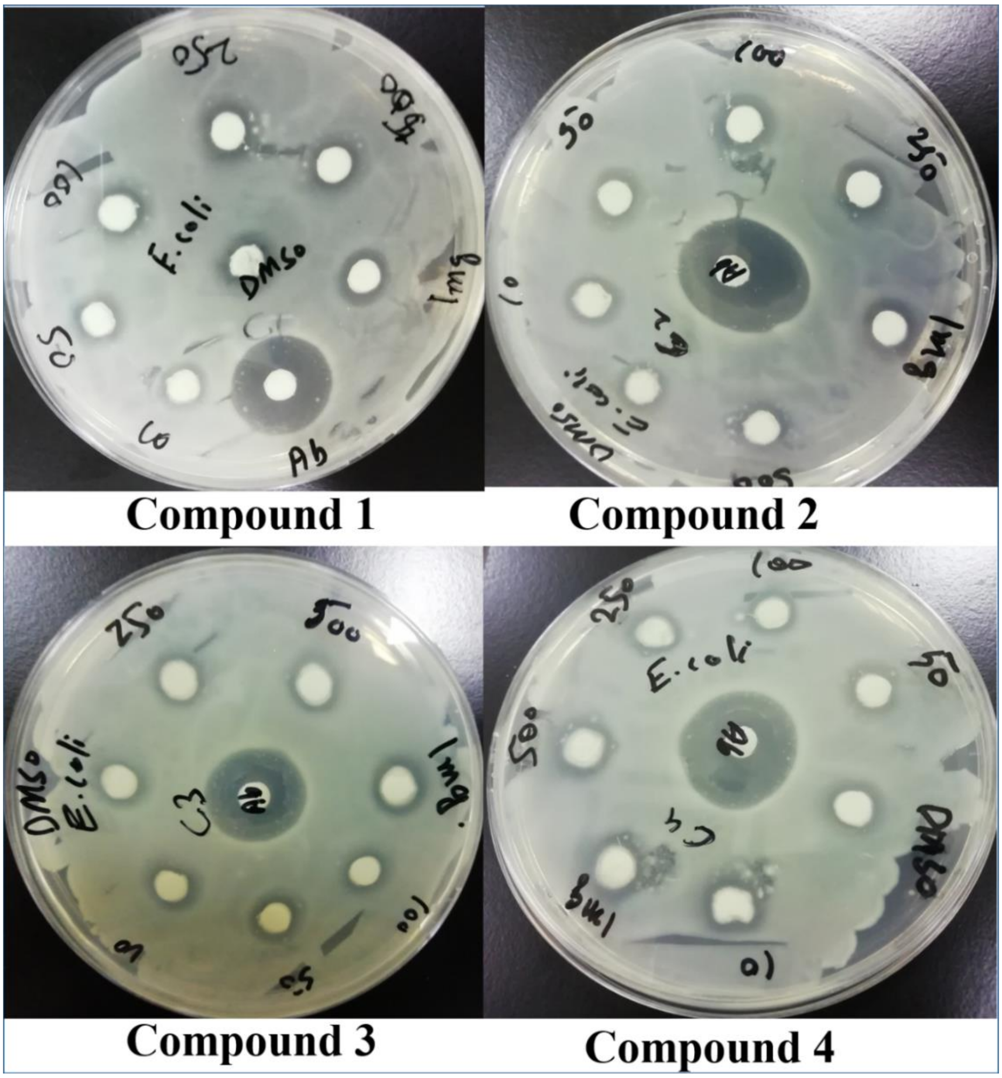

Figure S1. Antibacterial activity of the isolated compounds measured as disc diffusion.

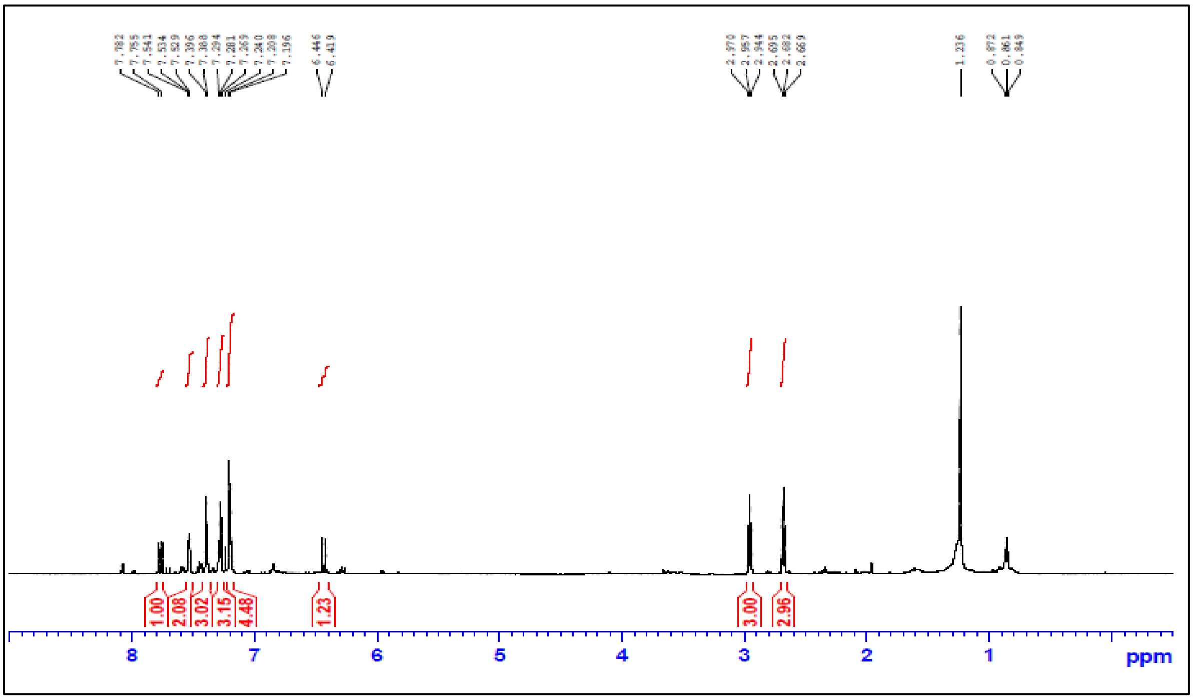
7.782
7.755
7.541
7.534
7.529
7.396
7.388
7.294
7.281
7.269
7.240
7.208
7.196
6.446
6.419
2.970
2.957
2.944
2.695
2.682
2.669
1.236
0.872
0.861
0.849
1.00
2.08
3.02
3.15
4.48
1.23
3.00
2.96
8
7
6
5
4
3
2
1
ppm

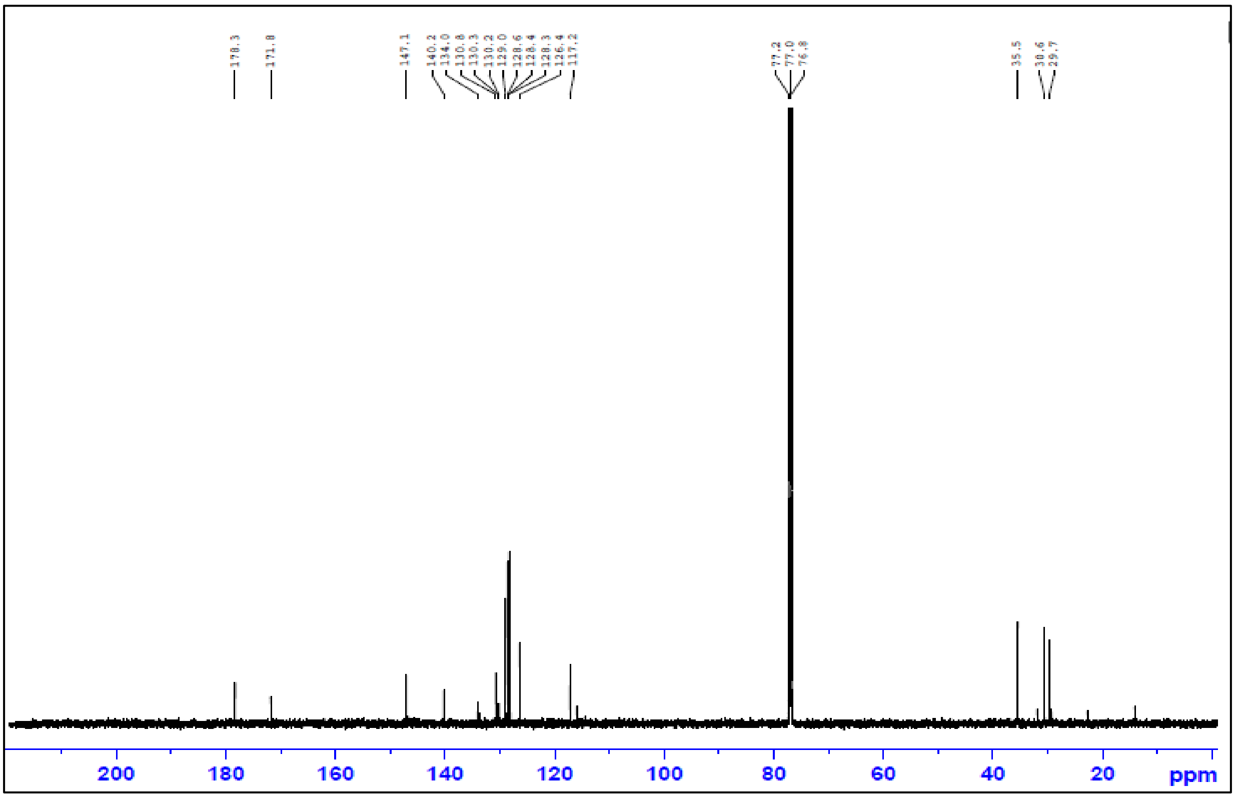
178.3
171.8
147.1
140.2
134.0
130.8
130.3
130.2
129.0
128.6
128.4
128.3
126.4
117.2
77.2
77.0
76.8
35.5
30.6
29.7
200
180
160
140
120
100
80
60
40
20
ppm

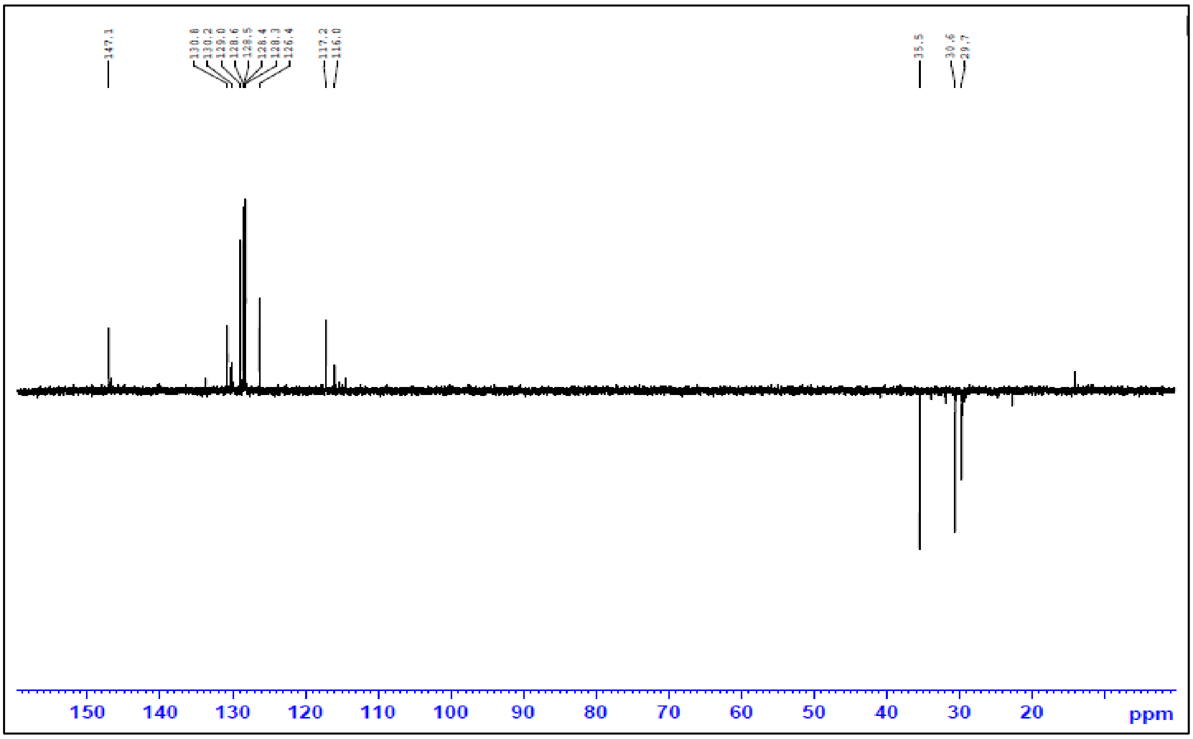
147.1
130.8
130.2
129.0
128.6
128.5
128.4
128.3
126.4
117.2
116.0
35.5
30.6
29.7
150
140
130
120
110
100
90
80
70
60
50
40
30
20
ppm

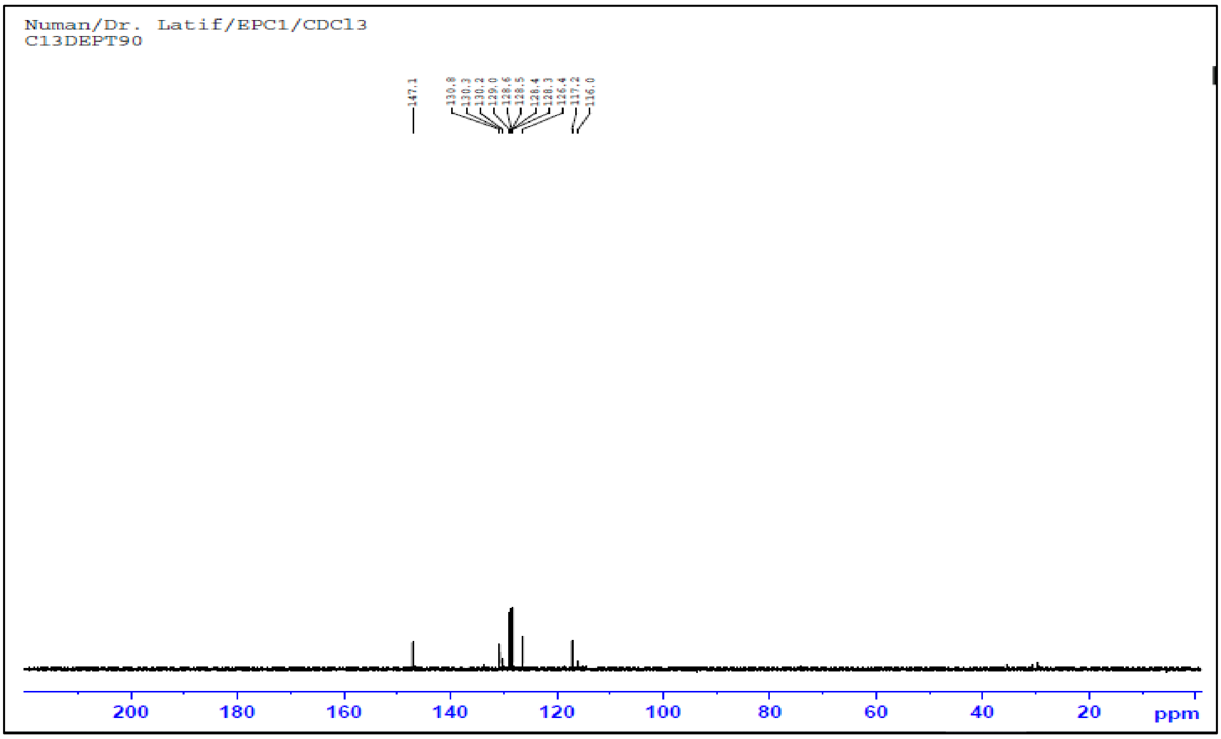
Numan/Dr. Latif/EPC1/CDCl3
C13DEPT90
147.1
130.8
130.3
130.2
129.0
128.6
128.5
128.4
128.3
126.4
117.2
116.0
200
180
160
140
120
100
80
60
40
20
ppm

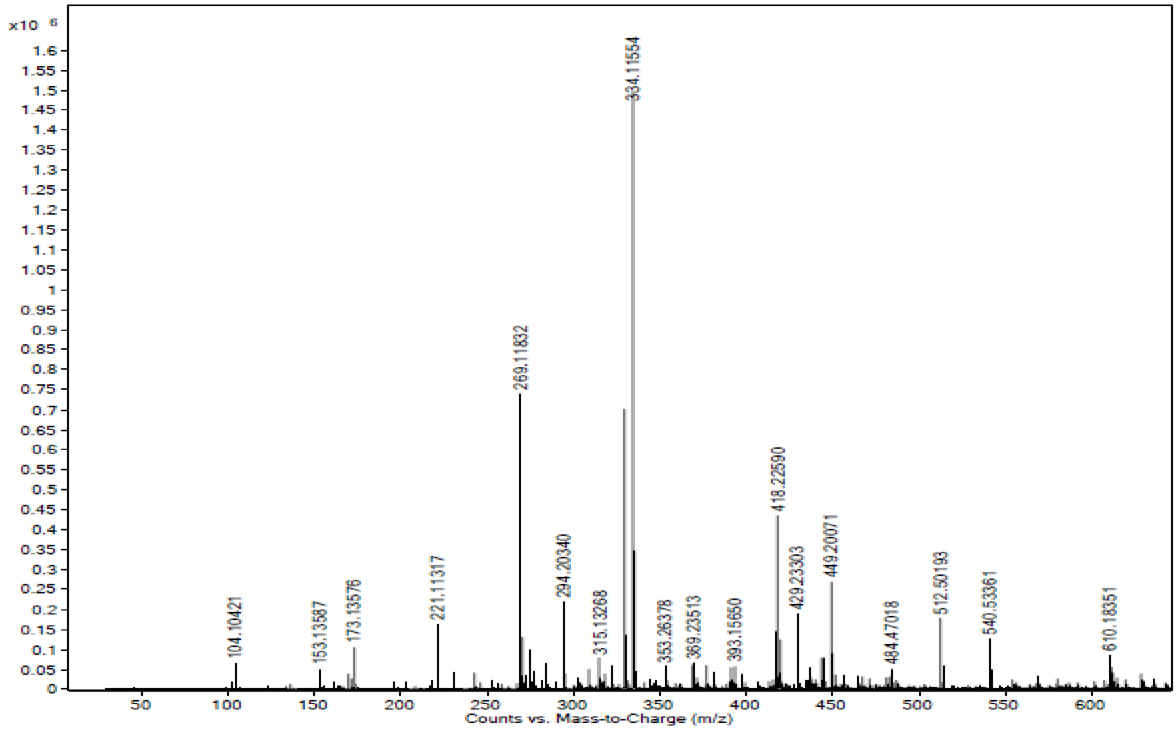

Figure S2: ^{1}H-, ^{13}C-NMR and HR-ESI-MS spectrum of **1**

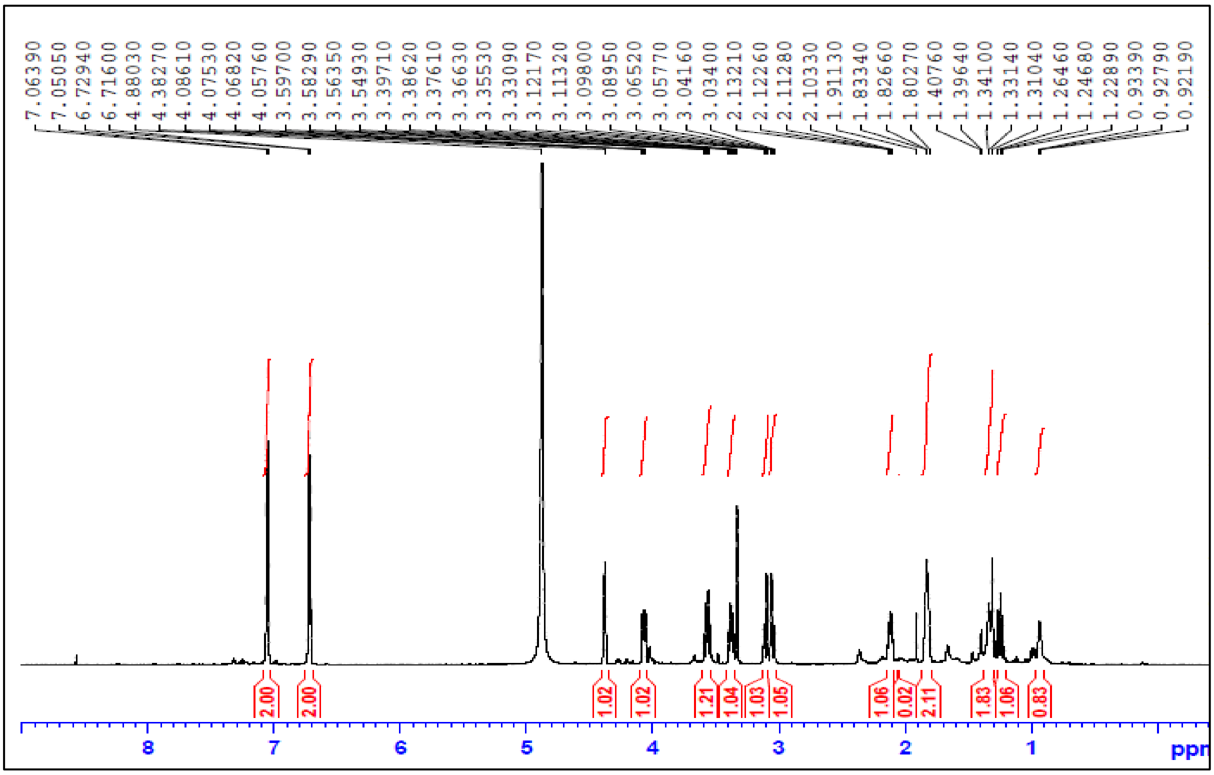

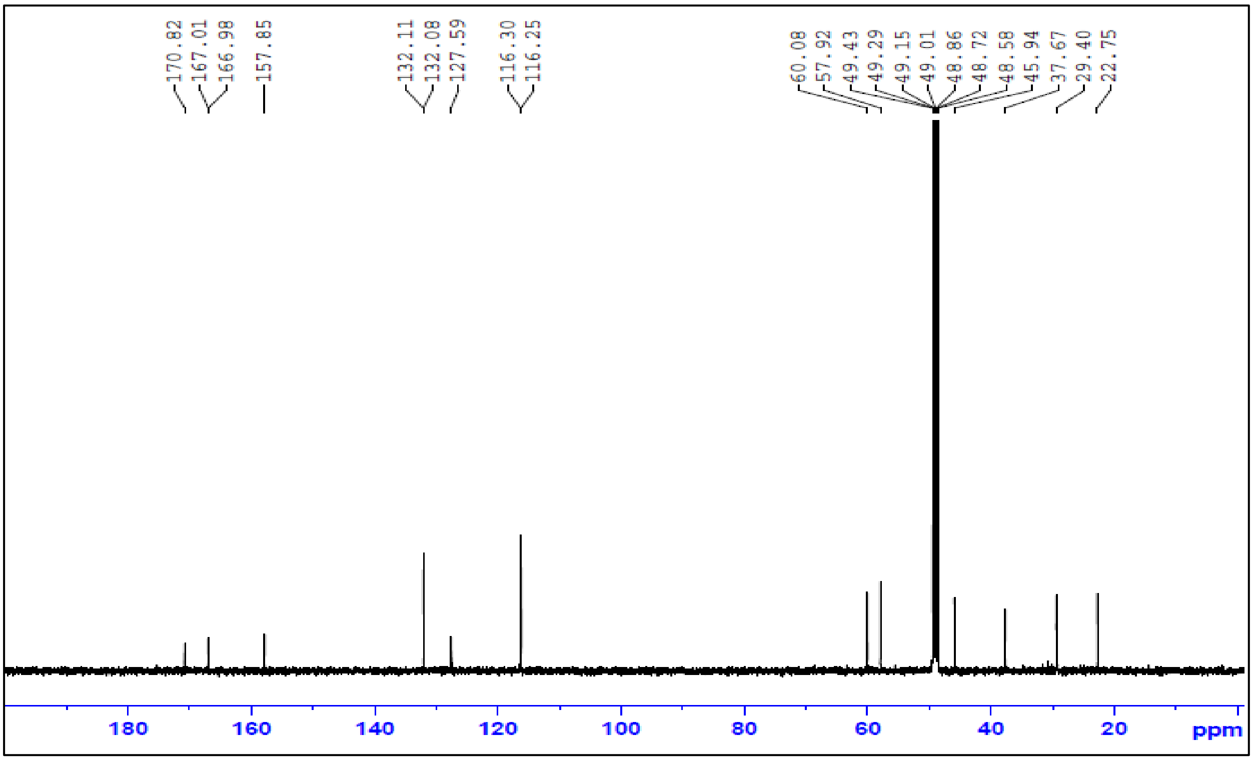

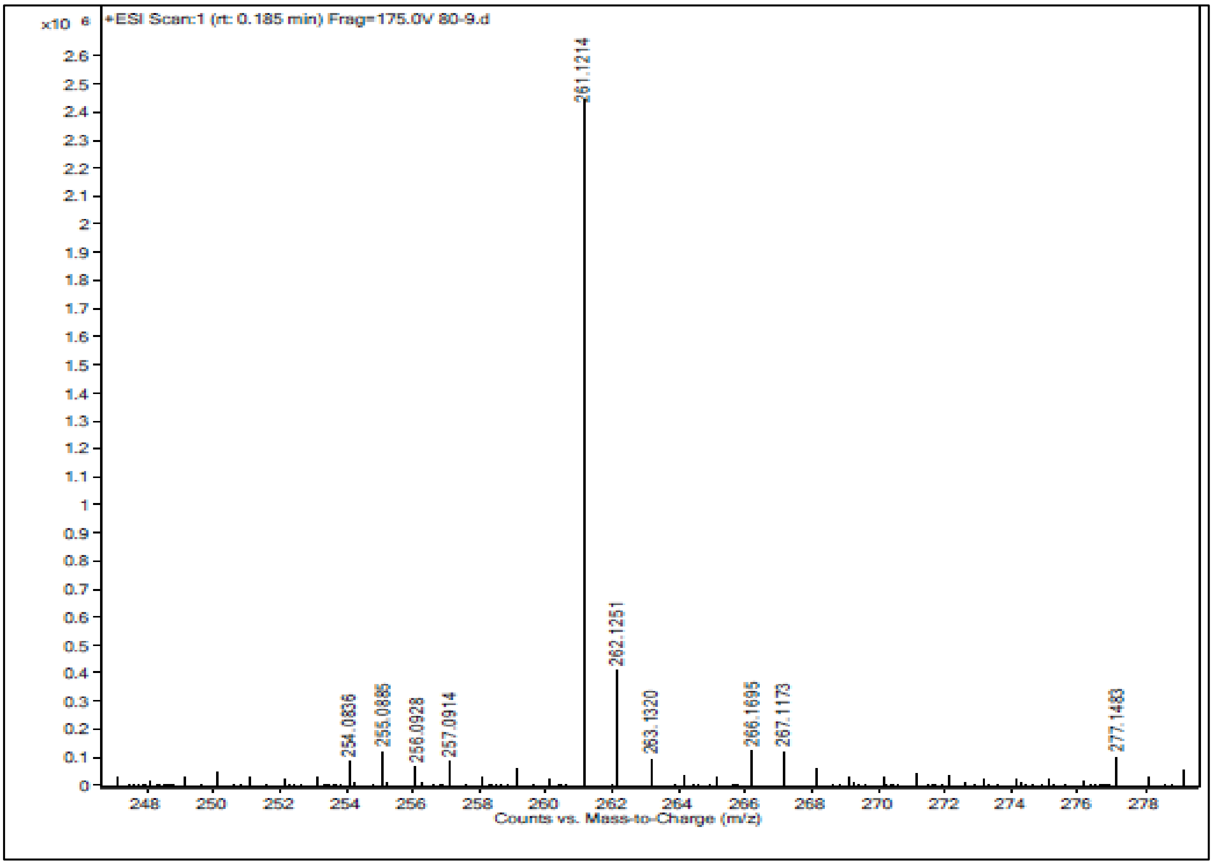

Figure S3: ^{1}H-, ^{13}C-NMR and HR-ESI-MS spectrum of **2**

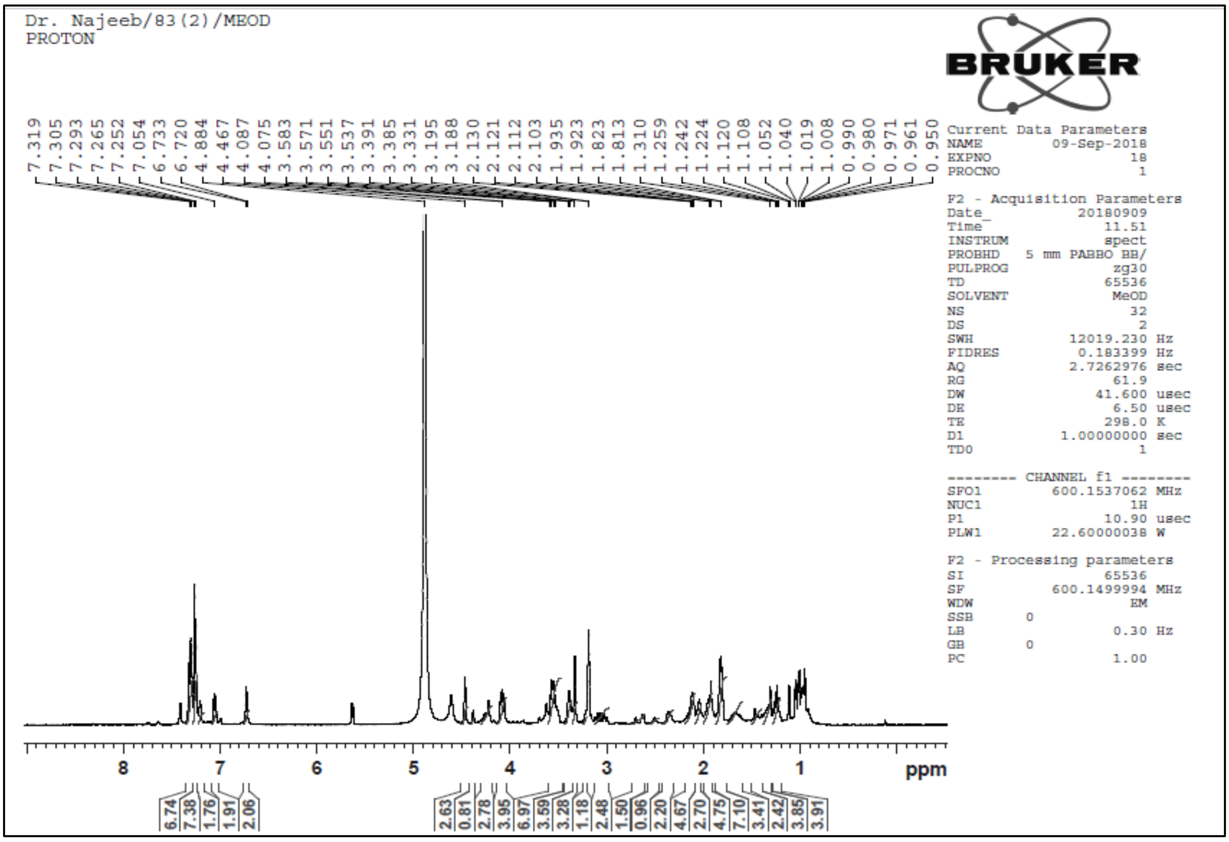
Dr. Najeeb/83(2)/MEOD
PROTON
BRUKER
7.319 7.305 7.293 7.265 7.252 7.054 6.733 6.720 4.884 4.467 4.087 4.075 3.583 3.571 3.551 3.537 3.391 3.385 3.331 3.195 3.188 2.130 2.121 2.112 2.103 1.935 1.923 1.823 1.813 1.310 1.259 1.242 1.224 1.120 1.108 1.052 1.040 1.019 1.008 0.990 0.980 0.971 0.961 0.950
Current Data Parameters
NAME 09-Sep-2018
EXPNO 18
PROCNO 1
F2 - Acquisition Parameters
Date_ 20180909
Time 11.51
INSTRUM spect
PROBHD 5 mm PABBO BB/
PULPROG zg30
TD 65536
SOLVENT MeOD
NS 32
DS 2
SWH 12019.230 Hz
FIDRES 0.183399 Hz
AQ 2.7262976 sec
RG 61.9
DW 41.600 usec
DE 6.50 usec
TE 298.0 K
D1 1.00000000 sec
TD0 1
======== CHANNEL f1 ========
SFO1 600.1537062 MHz
NUC1 1H
P1 10.90 usec
PLW1 22.60000038 W
F2 - Processing parameters
SI 65536
SF 600.1499994 MHz
WDW EM
SSB 0
LB 0.30 Hz
GB 0
PC 1.00
8 7 6 5 4 3 2 1 ppm
6.74 7.38 1.76 1.91 2.06
2.63 0.81 2.78 3.95 6.97 3.59 3.28 1.18 2.48 1.50 0.96 2.20 4.67 2.70 4.75 7.10 3.41 2.42 3.85 3.91

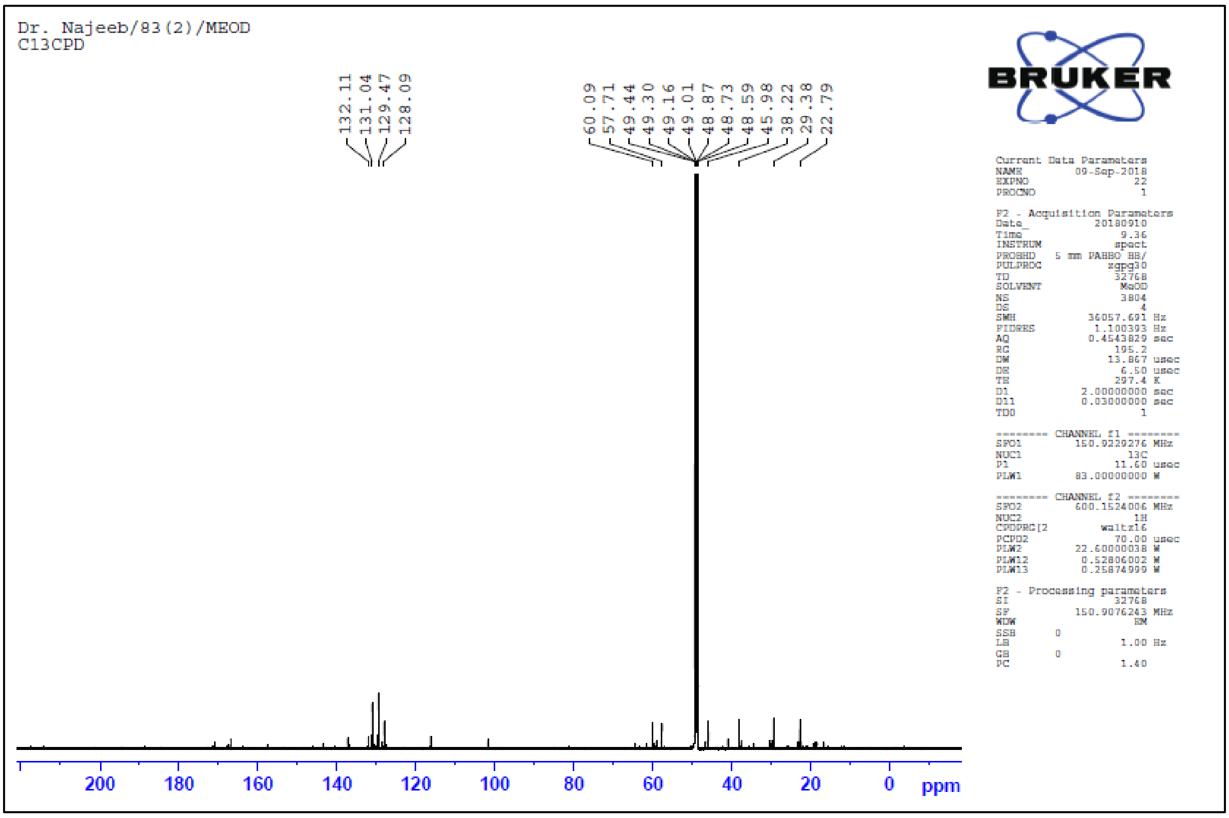
Dr. Najeeb/83(2)/MEOD
C13CPD
BRUKER
132.11 131.04 129.47 128.09
60.09 57.71 49.44 49.30 49.16 49.01 48.87 48.73 48.59 45.98 38.22 29.38 22.79
Current Data Parameters
NAME 09-Sep-2018
EXPNO 22
PROCNO 1
F2 - Acquisition Parameters
Date_ 20180910
Time 9.36
INSTRUM spect
PROBHD 5 mm PABBO BB/
PULPROG zgpg30
TD 32768
SOLVENT MeOD
NS 3804
DS 4
SWH 36057.691 Hz
FIDRES 1.100393 Hz
AQ 0.4543829 sec
RG 195.2
DW 13.867 usec
DE 6.50 usec
TE 297.4 K
D1 2.00000000 sec
D11 0.03000000 sec
TD0 1
======== CHANNEL f1 ========
SFO1 150.9229276 MHz
NUC1 13C
P1 11.60 usec
PLW1 83.00000000 W
======== CHANNEL f2 ========
SFO2 600.1524006 MHz
NUC2 1H
CPDPRG[2 waltz16
PCPD2 70.00 usec
PLW2 22.60000038 W
PLW12 0.52806002 W
PLW13 0.25874099 W
F2 - Processing parameters
SI 32768
SF 150.9076243 MHz
WDW EM
SSB 0
LB 1.00 Hz
GB 0
PC 1.40
200 180 160 140 120 100 80 60 40 20 0 ppm

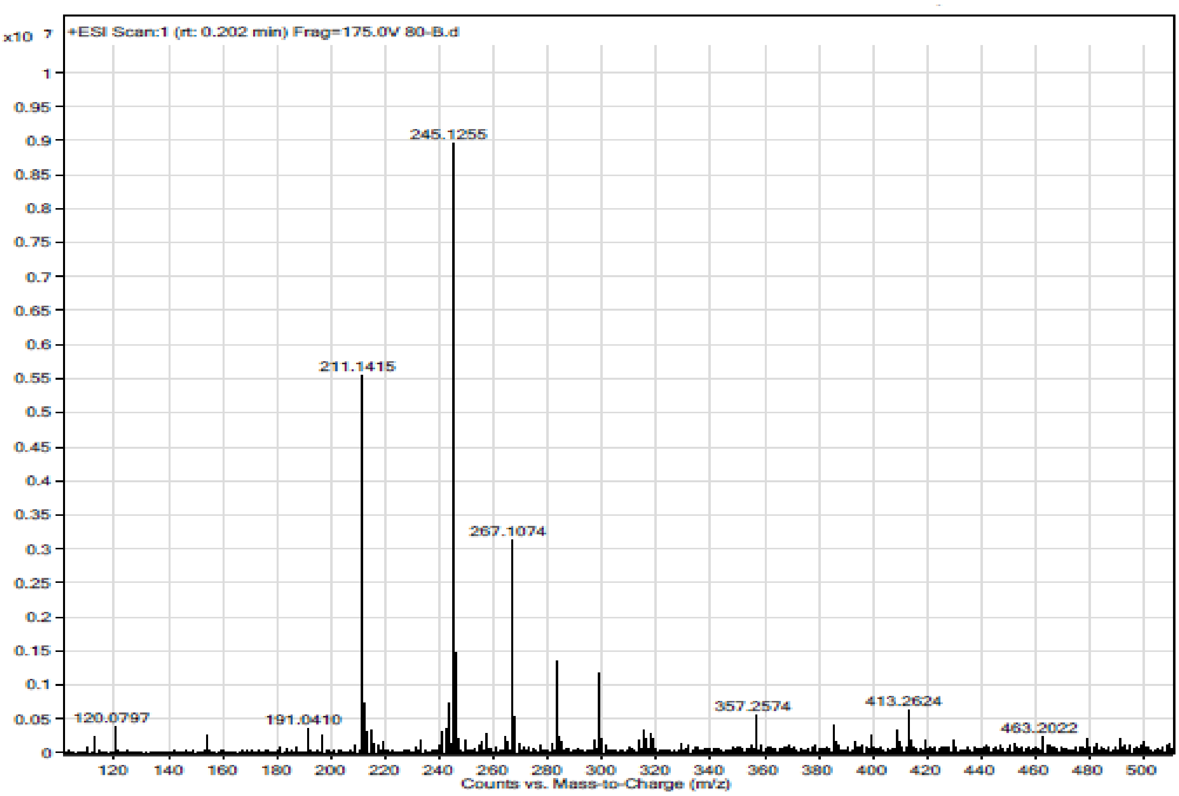

Figure S4: ^{1}H-, ^{13}C-NMR and HR-ESI-MS spectrum of **3**

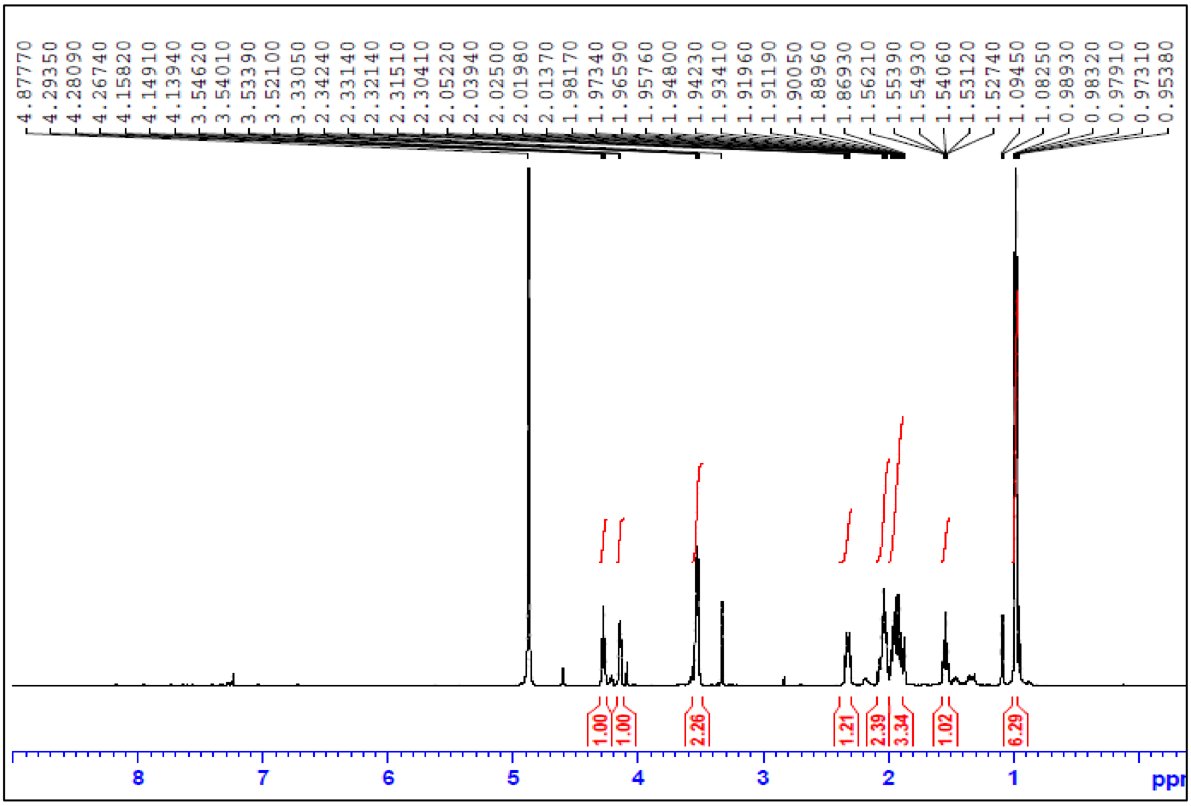

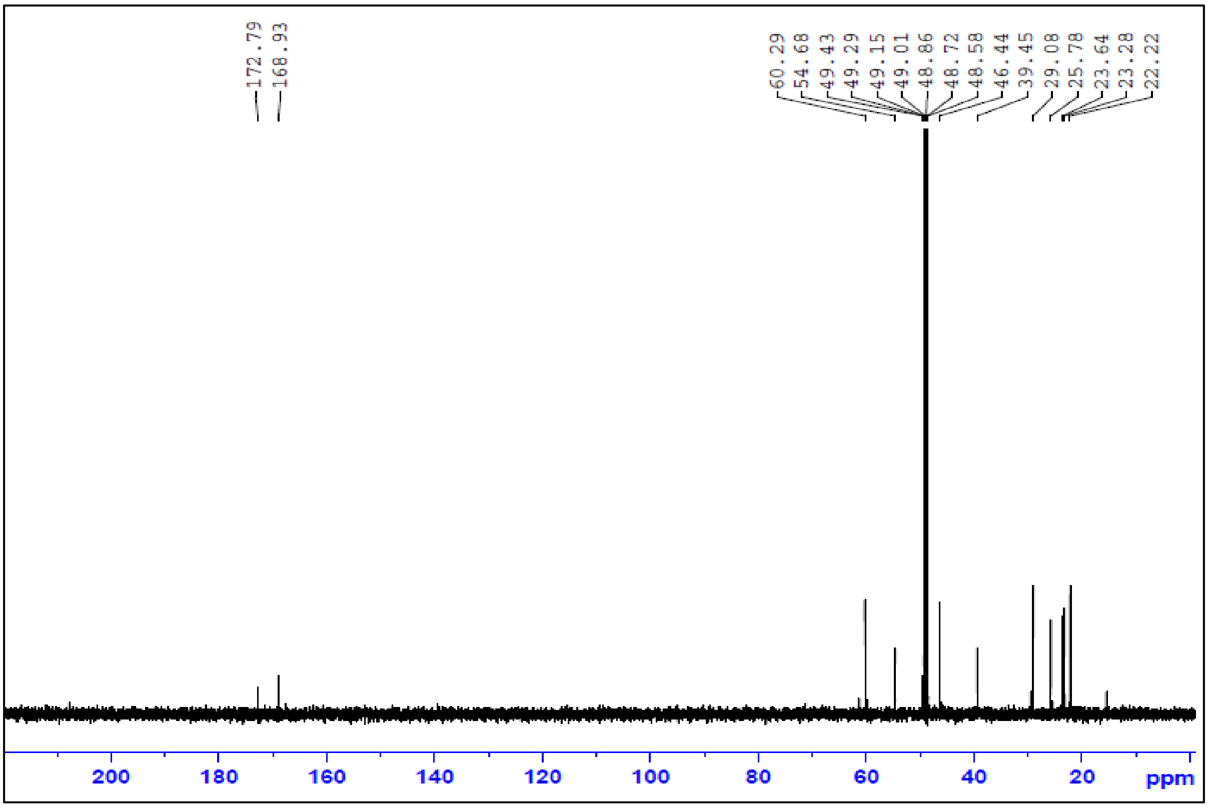

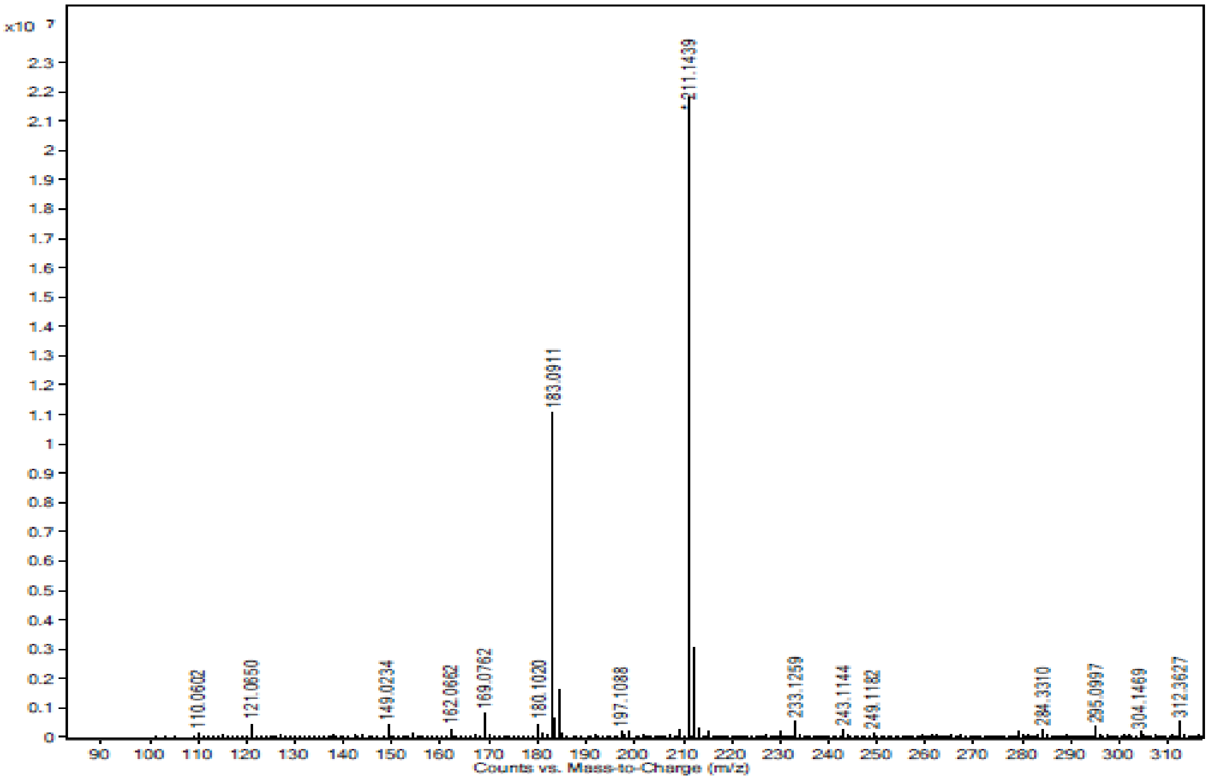

Figure S5: ^{1}H-, ^{13}C-NMR and HR-ESI-MS spectrum of **4**
